# Computer-Based Image Studies on Tumor Nests Mathematical Features of Breast Cancer and Their Clinical Prognostic Value

**DOI:** 10.1371/journal.pone.0082314

**Published:** 2013-12-12

**Authors:** Lin-Wei Wang, Ai-Ping Qu, Jing-Ping Yuan, Chuang Chen, Sheng-Rong Sun, Ming-Bai Hu, Juan Liu, Yan Li

**Affiliations:** 1 Department of Oncology, Zhongnan Hospital of Wuhan University, Hubei Key Laboratory of Tumor Biological Behaviors and Hubei Cancer Clinical Study Center, Wuhan, Hubei Province, China; 2 School of Computer, Wuhan University, Wuhan, Hubei Province, China; 3 Department of Breast and Thyroid Surgery, Renmin Hospital of Wuhan University, Wuhan, Hubei Province, China; Dana-Farber Cancer Institute, United States of America

## Abstract

**Background:**

The expending and invasive features of tumor nests could reflect the malignant biological behaviors of breast invasive ductal carcinoma. Useful information on cancer invasiveness hidden within tumor nests could be extracted and analyzed by computer image processing and big data analysis.

**Methods:**

Tissue microarrays from invasive ductal carcinoma (n = 202) were first stained with cytokeratin by immunohistochemical method to clearly demarcate the tumor nests. Then an expert-aided computer analysis system was developed to study the mathematical and geometrical features of the tumor nests. Computer recognition system and imaging analysis software extracted tumor nests information, and mathematical features of tumor nests were calculated. The relationship between tumor nests mathematical parameters and patients' 5-year disease free survival was studied.

**Results:**

There were 8 mathematical parameters extracted by expert-aided computer analysis system. Three mathematical parameters (number, circularity and total perimeter) with area under curve >0.5 and 4 mathematical parameters (average area, average perimeter, total area/total perimeter, average (area/perimeter)) with area under curve <0.5 in ROC analysis were combined into integrated parameter 1 and integrated parameter 2, respectively. Multivariate analysis showed that integrated parameter 1 (*P* = 0.040) was independent prognostic factor of patients' 5-year disease free survival. The hazard risk ratio of integrated parameter 1 was 1.454 (HR 95% CI [1.017–2.078]), higher than that of N stage (HR 1.396, 95% CI [1.125–1.733]) and hormone receptor status (HR 0.575, 95% CI [0.353–0.936]), but lower than that of histological grading (HR 3.370, 95% CI [1.125–5.364]) and T stage (HR 1.610, 95% CI [1.026 –2.527]).

**Conclusions:**

This study indicated integrated parameter 1 of mathematical features (number, circularity and total perimeter) of tumor nests could be a useful parameter to predict the prognosis of early stage breast invasive ductal carcinoma.

## Introduction

Breast cancer (BC) is the most common malignant tumor among females in both developed and developing countries [1]. The incidence is still on steady and rapid rising, and increasing number of younger patients with early BC are diagnosed every year [2]–[4]. For early BC, comprehensive treatments based on breast-conserving operation have been the major treatment modality [5], [6]. Although these patients just have a tumor mass, with neither lymph nodes involvement nor distant metastasis, they still face the potential risk of cancer recurrence and metastasis even after the completion of “clinically curative therapies” [7]. Therefore, it is imperative to predict the risk of cancer invasion and recurrence at the earliest possible time.

Currently, Tumor-node-metastasis (TNM) staging system is the universal language to determine the clinical stages of cancer and to predict the prognosis, and to guide the treatment options [8]. For early BC with N and M information both negative, this staging system is no longer efficient as a reliable tool to predict prognosis and to guide treatment option. At present, Worth Health Organization (WHO) histological grading (Nottingham modification of the Bloom-Richardson system or Scarff-Bloom-Richardson system) is the most widely accepted tool to judge tumor behaviors purely form the information on the tumor itself [9]. This grading system takes into consideration of three major histological and cytological features of BC, i.e., the tubule formation, nuclear pleomorphism and mitotic activity of cancer cells [10]. This classification system is well correlated to prognosis, however, it is highly experience-based, subjective, difficult for beginners, and the inter-observer variations among different pathologists are also major obstacles for a unified interpretation of the “same tumor mass” [11], [12]. As a result, the Breast Task Force of the American Joint Committee on Cancer excluded histological grading in its staging criteria because of “insurmountable inconsistencies” between institutions and pathologists [13]. Therefore, accurately predict the biological behaviors of BC based on the local tumor information and provide the basis and rationale for comprehensive treatments after breast-conserving operation still remains a major problem in the clinical diagnosis and treatment of BC.

Breast invasive ductal carcinoma (IDC) is the most common pathological type of BC [14], [15]. IDC is characterized by the formation of cancer cell groups with various geometrical and morphological features called tumor nests (TNs), which have lost structural consistency and functional coordination with the surrounding normal tissues [16]. TNs invade and destroy breast parenchyma and surrounding tissues leading to cancer spreading. From the viewpoint of tumor histopathology, the invasiveness of BC is mainly the collected behaviors of TNs rather than the individual cancer cells. Indeed, exploratory studies on the invasion patterns of breast and gastric cancers [17], and on the collective invasion behaviors of breast cancer cells [18] suggest that cancer invasion is largely the group behavior of cancer cells, i.e. TNs. Moreover, other studies on the morphological features of TNs also suggest the significance of TNs morphology in predicting the behavior and outcome of BC [11], [19]. Therefore, there must be rich information on cancer invasiveness hidden in the TNs beyond the scope of mere tumor grading analysis. So, systematical studies on the morphologic characteristics of TNs to dig out the hidden information on BC invasive behaviors are rational and essential to generate future prediction tools on the malignant biological behaviors of BC.

Histopathological tissue section is the most widely used material to study TNs morphologic characteristics. In current clinical practice, however, it is difficult to perform manual analysis because of the enormous amount information contained in the TNs in the tissue section. It is gratifying that computer automatic analysis technology has a significant advantage in dealing with this kind of big data information. Recently, several studies have developed various computer aided analysis methods to study the tumor image features [20]. Accurate identification of TNs boundary is the important premise of computer analysis [21], [22]. In our previous study, cytokeratin (CK), a kind of epithelial-specific marker was used to label BC TNs well [23]. The specific staining of CK in BC can clearly show the tumor boundary well which is most helpful to automatic recognition by computer system.

This study aimed to establish a computer image analysis and processing method to extract mathematical parameters (MPs) of breast IDC TNs, which were labeled with CK by immunohistochemistry (IHC). Using the clinical outcome information as the ultimate judgment, the prognostic value of these morphologic features was analyzed.

## Materials and Methods

### Patients and specimens

**Figure 1:**
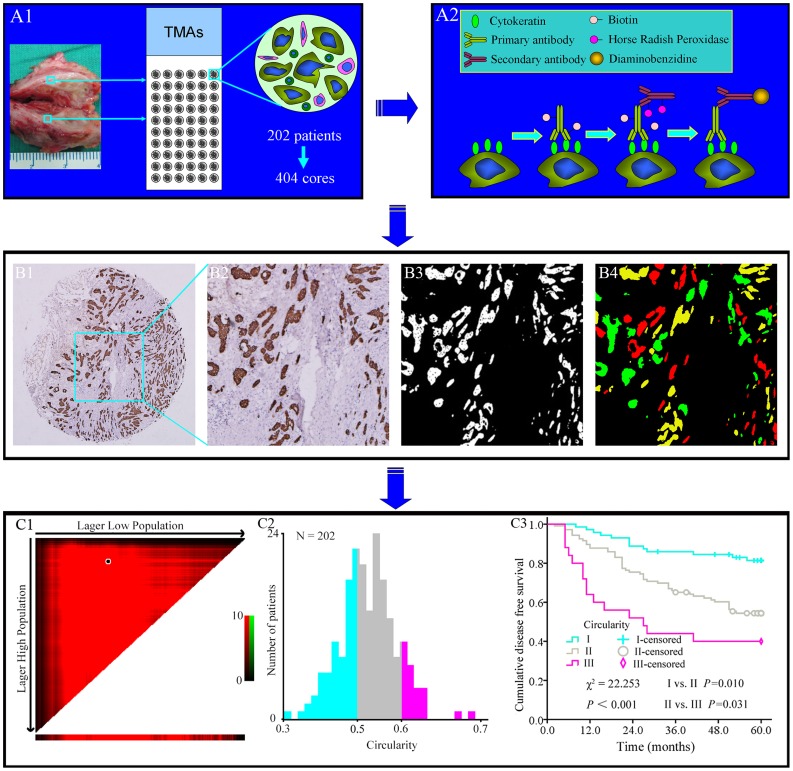
The design and major technical procedures of this study. Panel A1, 202 cases of breast IDC specimens were constructed into TMAs with 404 cores. Panel A2, CK was imaged based on IHC method. Panels B1 → B4, images processing and analysis. B1 → B2: Local amplification of IHC image; B2 → B3: After opening operation, flood-fill operation wad is performed to restore the entire morphological integrity of the IHC images, and then the Sobel algorithm was used to extract the boundary;B3 → B4: Correct initial segmentation image boundary, extract potentially useful information and delete noise signals by an expert pathologist-aided interactive method. Panel C1 → C3, MPs of TNs were divided into three grades by X-tile software. (MPs: mathematical parameters; TNs: tumor nests).

At our cancer center we have established a comprehensive cancer database, including BC. The collection of database and construction of tissue microarrays (TMAs) were based on clearly set criteria, and complete clinico-pathological information on the patients was available. This database has been the source of information for several published studies [24]–[26]. Based on this database, 202 cases breast IDC specimens were selected from the prepared TMAs (404 cores, 1.5 mm each core). Major clinico-pathological information was summarized in Table 1. TNM staging and histological grading were determined according to the 7th edition UICC TNM system [27] and WHO histological grading [9]. The study protocol was approved by the Institutional Ethics Committee of Zhongnan Hospital of Wuhan University. The study was undertaken according to the ethical standards of the World Medical Association Declaration of Helsinki. The whole analysis procedure was illustrated in Fig. 1.

**Table 1:** Major clinico-pathologic features of 202 patients.

Items	Value (%)
Age (years)	
≤50	122 (60.4)
>50	80 (39.6)
T stage	
T1 (diameter ≤2 cm)	26 (12.9)
T2 (2 cm<diameter ≤5 cm)	139 (68.8)
T3 (diameter >5 cm)	37 (18.3)
N status	
Negative	88 (43.6)
Positive	114 (56.4)
Histological grade	
I	29 (14.3)
II	125 (61.9)
III	48 (23.8)

### IHC staining of CK

Conventional streptavidin-peroxidase IHC method was used to stain TMAs. Major procedures of IHC were described previously and briefed as the following [28]. Firstly, the TMAs were heated at 60°C for 2 h in an oven and immersed in dimethylbenzene for 15 min to de-wax. TMAs were subjected to microwave antigen retrieval for 20 min at moderate baking temperature in 0.01 mmol/L (pH = 6.0) citrate buffer solution. After cooling at room temperature, TMAs were treated with 0.03% hydrogen peroxide methanol for 10 min to inactivate endogenous peroxidase. Then 2% bovine serum albumin (BSA) was used to block TMAs to decrease background intensity. Every slide was treated overnight at 4°C with 250 µL of mouse anti-human CK monoclonal antibody (AE1/AE3, dilution 1∶100, ZSGB-BIO, Beijing, China) and then incubated with the corresponding secondary antibody (dilution 1∶250) for 30 min at 37°C. The TMAs were treated with 0.2% diaminobenzidine (DAB, DAKO, Denmark) solution for 2 min to develop, counterstained with hematoxylin and differentiated by hydrochloric acid alcohol.

### Image acquisition and computer analysis

TMAs images with 2781×2781 pixels, 24 bit, and true color BMP format were obtained under Olympus BX51 microscope equipped with an Olympus DP72 camera (Olympus Optical Co., Ltd. Tokyo, Japan) at 40× magnifications. Theoretically speaking, in order to obtain morphological characteristics of each TN, every TN should be clearly demarcated and their exact boundaries should be identified. In real-life situation, however, it is difficult to set clear-cut margins on all IHC images, because of the inherent inability of the current imaging analysis software on the one hand and the technical limitations of IHC to achieve completely satisfactory staining results on the other hand. Therefore, in our analysis, we adopted a computer automatic recognition supplemented with expert-pathologist (JPY) aided discrimination strategy, so as to maximally differentiate any obscure boundaries. To achieve this objective, we developed a pixel-based interactive segmentation strategy for images with optimal IHC staining, involving the following four steps. First contrast enhancement and de-noising methods were adopted to improve the quality of the obtained images. Second, a self-adaptive Otsu threshold method was adopted to convert the obtained images into binary images. Third, morphological opening methods were used to smooth the silhouette of all images, separate the fine boundaries between the adjacent images, and eliminate any possible protrusions due to artifact in IHC staining. Fourth, flood-fill operation was conducted to automatically restore the entire morphological integrity of the IHC images, and all the boundary information was extracted using Sobel algorithm. For images with sub-optimal IHC staining results, an expert pathologist-aided interactive method was adopted to extract potentially useful information and delete noise signals.

### Definition and grading of MPs

After the borderline of TNs was set accurately by computer, 8 MPs characterizing the TNs were automatically calculated by the computer image analysis and processing method. The terminology and definition of these 8 MPs are: (1) Number, defined as the total number of TNs in one core of the TMAs; (2) Total perimeter, defined as the sum perimeter of each TN and expressed as pixels; (3) Average perimeter, calculated by total perimeter divided by the number of TNs, and expressed as pixels; (4) Total area, defined as the sum area of all the TNs in one core of TMAs, and expressed as pixels; (5) Average area, calculated by total area divided by the number of TNs, and expressed as pixels; (6) Total area/total perimeter ratio, defined as a ratio of total area of TNs divided by the total perimeter; (7) Average (area/perimeter) ratio, defined as the sum of area/perimeter ratio of each TN divided by the number of TNs, the equation is  =  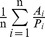; and (8) Circularity, defined as the average of the sum of 4π times area divided the square of perimeter, the equation is  =  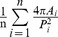, where P_i_ stands for perimeter of the ith TN, and A_i_ stands for area of the ith TN.

As these parameters were continuous variables, it is necessary to convert these measured variables to categorical variables for statistical analysis. For this purpose, the X-tile software [29] based on the best *P* value principle was adopted to automatically judge the cut-off points [30], [31] and divide each measured variable into three categories, which were attributed as grade I, grade II and grade III.

### Statistical analysis

Statistical analysis was performed with SPSS 17.0 software (SPSS Inc. Chicago, IL, USA). Five-year disease free survival (5-DFS) was calculated by the Kaplan-Meier method and analyzed by the Log-rank test. Correlation test was calculated by Pearson chi-square. Receiver operating characteristic (ROC) curve analysis was used to determine the predictive value of the parameters of interest. Multivariate survival analysis was performed with the Cox proportional hazards method. Two sided *P*<0.05 was considered as statistically significant.

## Results

### IHC staining results

**Figure 2:**
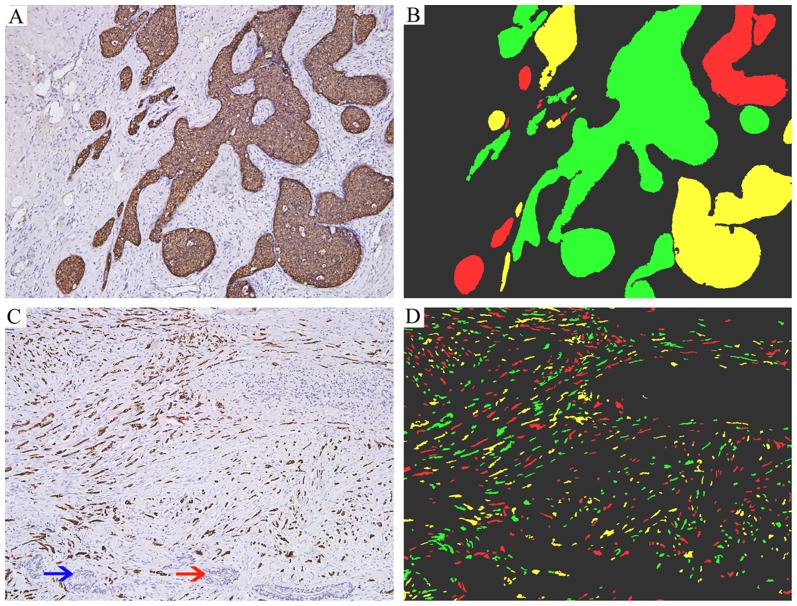
Typical IHC images and computer analysis results. Panels A and C: P-CK was specifically expressed in cancer cells in large TNs (A) or small TNs (C), but not expressed in stromal cells, blood vessel (red arrow), or nerves (blue arrow). Panels B and D: The TNs information revealed by IHC staining was extracted and converted into corresponding color information by computer.

CK is mainly expressed in membrane and cytoplasm of BC cells, but not expressed in stromal cells. There was no background “noise” in the IHC staining, which ensures good image quality for computer analysis. Typical IHC staining images and computer analysis results were shown in Fig. 2.

### MPs of the TNs and grading results

The X-tile software based on the best *P* value principle was adopted to automatically judge the cut-off points of 8 MPs and divide each measured variable into three categories, which were attributed as grade I, grade II and grade III. The range, cut-off points and results of grading of 8 MPs were showed in Table 2.

**Table 2:** MPs of breast IDC nests determined by computer recognition and analysis.

Parameters	Results	Cut-off points by X-tile		Grading (n)		
	Median (range)	Cut-off points I/II	Cut-off points II/III	I	II	III
Number^1^	320.5 (15.50–1636.50)	253.00	834.00	80	101	21
Total perimeter^2^	113064.70(1647.42–308789.00)	89846.85	145075.00	77	77	48
Average perimeter^2^	324.99 (86.81–2268.65)	310.09	434.31	96	41	65
Total area^2^	1353878.00 (9821.00–1822770.00)	566207.00	1661470.00	27	105	70
Average area^2^	4238.22 (407.57–121350.40)	1609.78	8882.37	50	93	59
Total area/total perimeter ratio^1^	12.26 (4.22–69.14)	7.95	21.63	54	102	46
Average(area/perimeter) ratio^1^	5.76 (3.00–19.67)	4.36	6.61	32	102	68
Circularity^1^	0.50 (0.32–0.74)	0.48	0.57	71	106	25

**Footnotes:** ^1^: no units; ^2^: pixel.

### Correlation analysis of TNs MPs and 5-DFS

**Figure 3:**
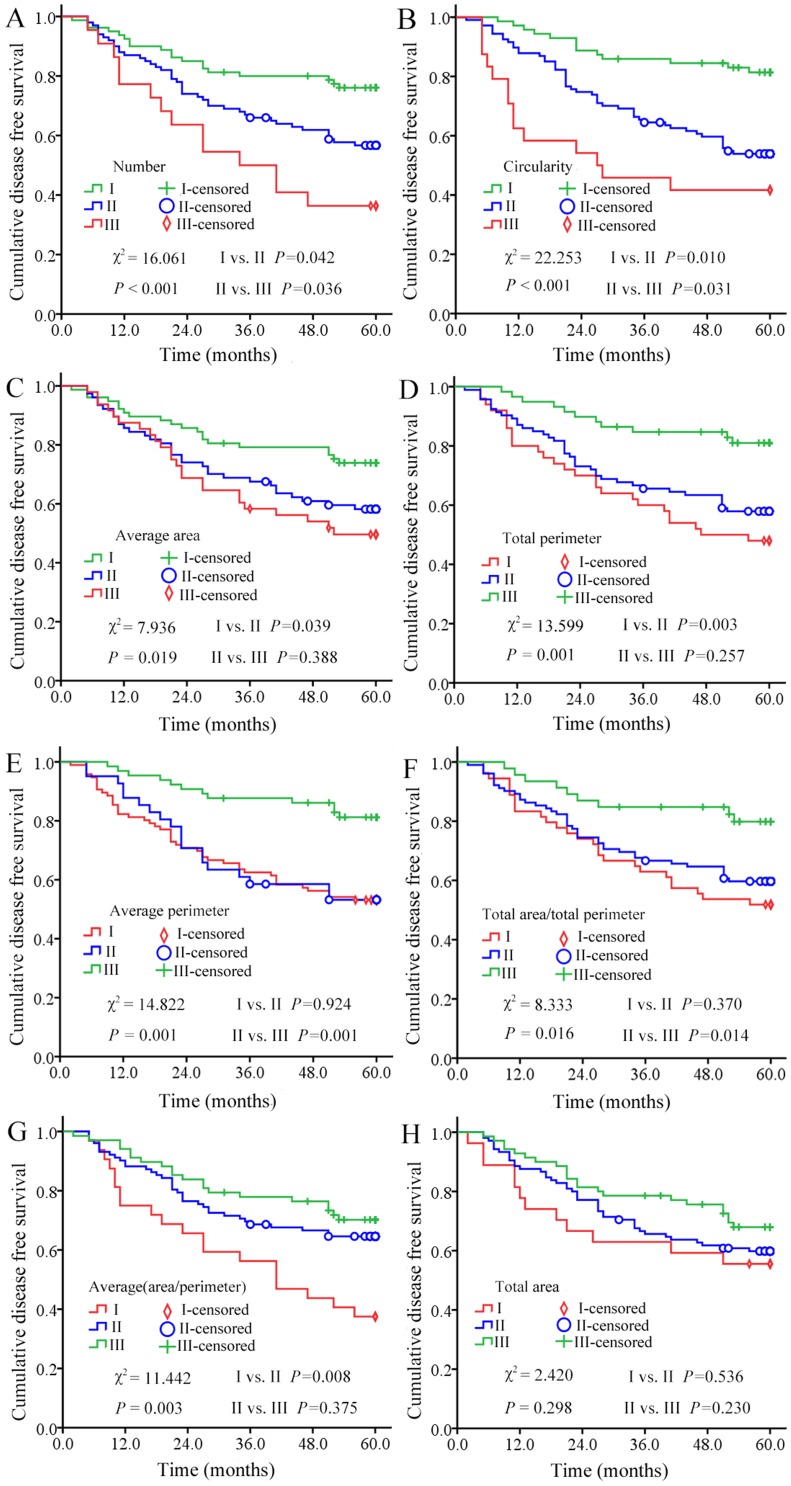
The relationship between MPs of TNs and 5-DFS of patients. Among the 8 MPs studied, 3 MPs (number, circularity and total perimeter, in panels A, B and C) negatively correlated with 5-DFS, 4 MPs (average area, average perimeter, total area/total perimeter ratio, and average (area/perimeter) ratio, in panels D, E, F and G) positively correlated with 5-DFS, and only 1 parameter (total area, in panel H) not correlated with 5-DFS.

Correlation analysis was done to investigate the relationship between MPs of TNs and 5-DFS of the patients. As shown by the Kaplan-Meier survival curve (Fig. 3), the following 7 TNs MPs had statistically significant correlations with 5-DFS of patients: number, average area, total perimeter, average perimeter, total area/total perimeter ratio, average (area/perimeter) ratio and circularity. Two parameters, number and circularity could distinguish 5-DFS into three classes which had statistical differences in each of them (Fig. 3A, 3B). In contrast, the other five parameters could only divide 5-DFS of patients into two classes with statistically significant differences (Fig. 3C, 3D, 3E, 3F, 3G). There was no significant correlation between the total area of TNs and 5-DFS (Fig. 3H).

### ROC analysis on MPs and traditional parameters

**Figure 4:**
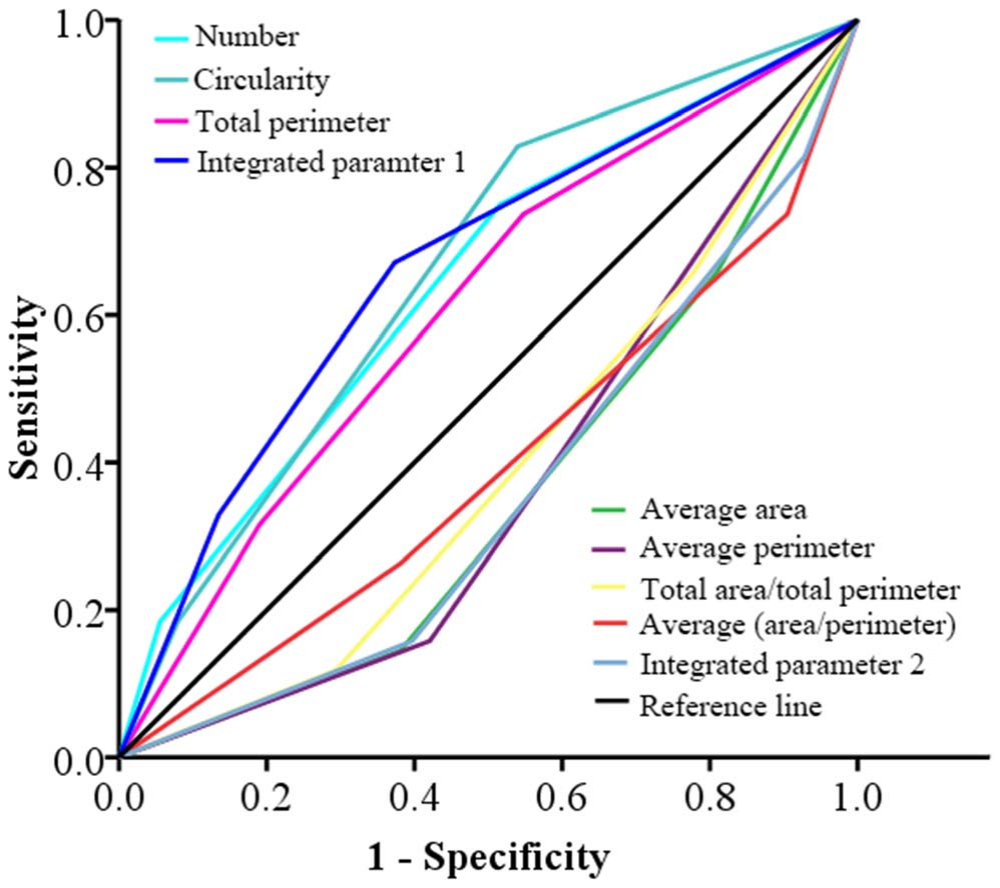
ROC analysis of the predictive value for recurrence. Among the 7 MPs, 3 parameters (number, circularity and total perimeter) and integrated parameter 1 had AUC>0.5, the other 4 parameters (Average area, average perimeter, total area/total perimeter and average (area/perimeter)) and integrated parameter 2 had AUC<0.5.

To proceed to a deeper analysis, we conducted ROC study to analyze 7 MPs which had statistical significance in Kaplan-Meier analyses (Fig. 4). The ROC curve showed that 3 MPs (number, circularity and total perimeter) with areas under the curve (AUC)>0.5 negatively correlated with prognosis, and the other 4 MPs (average area, average perimeter, total area/total perimeter, average (area/perimeter)) with AUC<0.5 positively correlated with prognosis (Fig. 4). All the 7 MPs had statistical correlation with 5-DFS (*P*<0.05, Table 3). Then, the 3 negative MPs and 4 positive MPs were given corresponding scores according to their grades (1 score for grade I, 2 scores for grade II, and 3 scores for grade III) and combined into integrated parameter 1 and integrated parameter 2, respectively. Then, integrated parameter 1 and integrated parameter 2 were divided into 3 tiles (low, middle and high tiles) by X-tile software. The result of ROC analysis showed that integrated parameter 1 had AUC higher than any of the 3 MPs, and integrated parameter 2 had AUC lower than any of the other 4 MPs (Table 3).

**Table 3:** Results of ROC analysis on 7 MPs, integrated parameters 1 and 2.

Parameters	Area	95% CI	P value
Number	0.644	0.565–0.722	0.001
Circularity	0.661	0.585–0.737	<0.001
Total perimeter	0.611	0.531–0.691	0.008
**Integrated parameter 1**	0.665	0.587–0.743	<0.001
Average area	0.357	0.280–0.434	0.001
Average perimeter	0.368	0.290–0.445	0.002
Total area/total perimeter	0.390	0.311–0.468	0.009
Average (area/perimeter)	0.395	0.313–0.477	0.012
**Integrated parameter 2**	0.355	0.278–0.432	0.001

### Multivariate analysis of integrated parameters 1 and 2, and traditional parameters

To further validate their prognostic significances, the integrated parameter 1, integrated parameter 2 and traditional prognostic parameters were subjected to Cox proportional hazard model analysis. Multivariate analysis identified 5 variables including integrated parameter 1 (*P* = 0.040), T stage (*P* = 0.038), N stage (*P* = 0.002), histological grade (*P* **<**0.001), and hormone receptor status (*P* = 0.026) were independent prognostic factors for 5-DFS; but integrated parameter 2 (*P* = 0.512) and Her-2 gene (*P* = 0.588) were not independent prognostic factors (Table 4).

**Table 4:** Multivariate analysis by Cox proportional hazard model.

Parameters	HR (95% CI)	*P* value
T stage	1.610 (1.026–2.527)	0.038
N stage	1.396 (1.125 –1.733)	0.002
Histological grade	3.370 (1.125–5.364)	<0.001
Integrated parameter 1	1.454 (1.017–2.078)	0.040
Integrated parameter 2	0.857 (0.540–1.360)	0.512
Hormone receptor status	0.575 (0.353–0.936)	0.026
Her-2 gene	1.152 (0.691–1.922)	0.588

The integrated parameter 1 was also compared with currently accepted histopathological prognostic factors. As shown in Table 4, the hazard risk ratio of integrated parameter 1 (HR 1.454, 95% CI [1.017–2.078]) was higher than N stage (HR 1.396, 95% CI [1.125 –1.733]) and hormone receptor status (HR 0.575, 95% CI [0.353–0.936]), but lower than the histological grading (HR 3.370, 95% CI [1.125–5.364]), and T stage (HR 1.610, 95% CI [1.026 –2.527]).

### Typical examples of impacts of integrated parameter 1 on clinical prognosis

**Figure 5:**
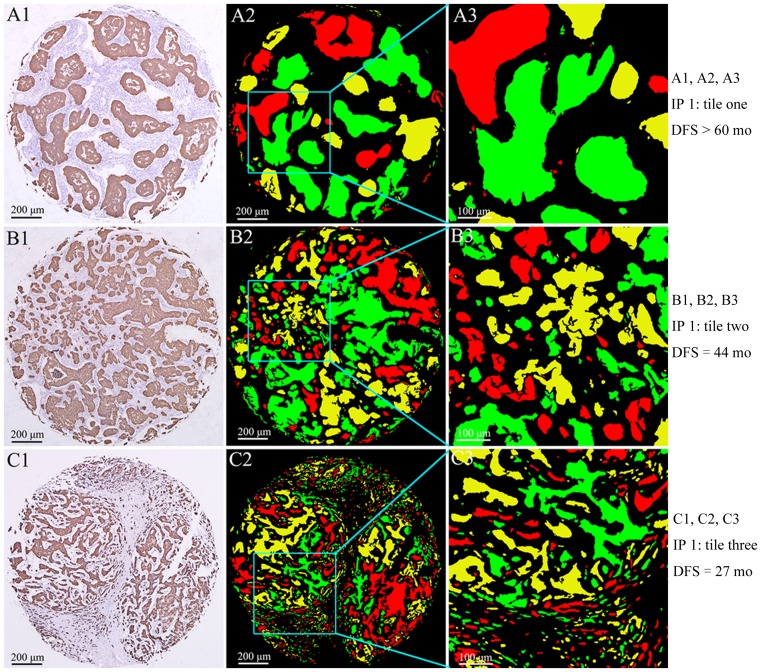
The impacts of different integrated parameter 1 tiles on 5-DFS. A1, B1 and C1 are the IHC images of integrated parameter 1 tiles one, two and three, respectively. A2, B2 and C2 are the corresponding results of computer analysis. A3, B3 and C3 are local amplification of computer analysis results. (IP: integrated parameter).

From the above analyses, we found that integrated parameter 1 was an independent prognostic factor. Integrated parameter 1 is the combination of TNs number, circularity and total perimeter. Patients with higher tile of integrated parameter 1 of TNs had a worse prognosis than those with lower tile. To further validate the prognostic significance of integrated parameter 1 well, we selected three typical examples of integrated parameter 1 tiles one, two and three. Patient with TNs integrated parameter 1 tile one had the best 5-DFS, patient with integrated parameter 1 tile three had the worst 5-DFS, and patient with circularity tile two had intermediate 5-DFS (Fig. 5).

## Discussion

The future direction of clinical oncology will be the transition from therapeutic oncology to preventive and predictive oncology. A series of individualized preventive and therapeutic measures based on tumor characteristics have been formulated in this regard [32]. The essence of predictive oncology is to predict future biological behaviors of cancer based on the information of local tumor, especially the risk of invasion and metastasis. To achieve this goal, more information on cancer invasion should be obtained from local tumor in addition to conventional pathological features. Since cancer invasion and metastasis are collective behaviors of cancer cell groups/TNs, endeavors to extract and decipher key information on TNs are rational strategy in this regard. If the novel parameters thus obtained could have significant impact on clinical prognosis, they could be selected as useful parameters in the construction of a new predictive model.

In the field of computer-aided automatic classification and prediction of cancer behaviors, a number of pioneering works have been reported (Table 5). Computer-based automatic analysis system can quickly extract large amount of information on tumor tissue sections, and help objective evaluation of cancer behaviors based on big data information. Appling such strategy on 576 invasive BC specimens conventionally processed by routine histopathological procedure, Beck et al [11] extracted 6,642 features by an image processing pipeline and found 3 stromal and 8 epithelial features were independently related to 5-year survival. Similarly, using computer software called support vector machine classifier to recognize and classify BC tissue sections after conventional hematoxylin and eosin (HE) stain, Yuan et al [12] systematically studied tumor stromal cells and lymphocytes based on nucleus morphology recognition, and found lymphocytes infiltration and spatial pattern of tumor stroma were independently related to prognosis of patients. These studies have pointed new directions for computer analysis on diagnosis and prediction of tumor behaviors. However, the technical shortcoming common in these two studies was the use of traditional HE staining, which due to its inherent technical limitations, suffers from relatively poor image quality, complex texture features and artifacts, therefore could challenge computer analysis and compromise the repeatability of the results.

**Table 5:** Computer study on grading and predicting tumor behavior.

Researches	Object	Major findings
Yuan et al[12], 2012	564 ER negative BC tissues	Support vector machine classifier divided tumor into cancer, lymphocytes, and stroma components; Lymphocyte infiltration index positively related to prognosis; Spatial pattern of stromal cells is an independent survival factor.
Beck et al[11], 2011	576 BC tissues	Image processing pipeline extracted 6642 features; 3 stromal and 8 epithelial features related to 5-year survival, significances of stromal were higher than epithelial.
Tambasco et al[19], 2010	379 breast IDC tissues	Computer fractal analysis technique extracted fractal dimension; Morphologic complexity (fractal dimension) of TNs negatively related to prognosis.
Basavanhally et al[36], 2013	126 ER positive BC tissues	Multi-field-of-view classifer extracted image features related to spatial arrangement of cancer neclei and textural patterns within nuclei to automatic classification; AUC value of the grading system distinguished low vs. high, low vs. intermediate, and intermediate vs. high grade were 0.93, 0.72 and 0.74.
Ozdemir et al[37], 2012	3236 colon images	A resampling-based Markovian model developed to automatic classification; Accuracy of 90.32% for automated classification on colon cancer.
Dundar et al[38], 2011	476 breast images	A prototype system for automatic classification developed to automatic classification; Accuracy of 87.9% for automatic classification on breast lesions.
Sertel et al[39], 2009	510 follicular lymphoma images	Model-based intermediate representation and color-texture analysis method developed to automatic classification; Overall accuracy of 85.5% for automatic classification on follicular lymphoma.
Altunbay et al[22], 2010	213 colon images	Delaunay triangulation, a method quantified the tissue by distribution of cell nuclei developed to automatic classification; Accuracy of 82.65% for automated classification on colon tissues.
Kong et al[40], 2009	33 neuroblastoma specimens	Multi-resolution framework was developed to automatic classification; Accuracy of 87.88% for automatic classification on neuroblastoma.
Tabesh et al[41], 2007	268 prostate cancer tissues	A sequential forward feature selection algorithm developed to automatic classification; Accuracy of 81.0% for automatic classification on prostate cancer.

To avoid such problems, specific staining of target tissue by IHC method has been used to increase the contrast between target and non-target areas, thus could make images more suitable for computer recognition than conventional HE images and improve the reliability of analysis results [19]. This strategy was used by Tambasco et al [19], who studied the fractal dimension of BC nests after IHC staining with pan-CK, analyzed the morphologic complex of IDC epithelial architecture, and found high fractal dimension was related to poor prognosis. In these studies, no-specific staining and other inherent defects in tumor tissues were all recognized as “targets” by computer automatic analysis. Although this method can extract information in tumor tissues quickly and objectively, computer automatic analysis system could not effectively differentiate no-target information, especially for sub-optimal images. So, appropriate pathologist-assisted operation is essential to extract tumor tissue information more accurately for sub-optimal images.

In this study, efforts were made to extract meaningful information on prognosis from TNs mathematical features, with the aid of computer based image recognition and analysis. Compared with other similar studies, we have made two technical improvements to optimize the precision and repeatability of the analysis. Firstly, the IHC stain with CK to mark the TNs significantly enhanced the contrast between TNs and the surrounding stroma, rendering the computer recognition much easier and with less error. Secondly, we adopted an expert-pathologist aided strategy to filter out the none-target information, making the information analysis more focused and reliable. After these improvements, we have obtained 8 MPs to characterize the size, shape, surface features, spatial closeness and discreteness of TNs. Using 5-DFS as golden criterion to judge their clinical significances, we found 7 out of these 8 parameters of TNs had statistically significant correlation with 5-DFS. The 3 MPs with AUC>0.5 and the other 4 MPs with AUC<0.5 in ROC analysis were combined into integrated parameter 1 and integrated parameter 2, respectively. Then we further analyzed the efficiency of these parameters by Cox multivariate analysis, which identified integrated parameter 1 of TNs as an independent prognostic factor for 5-DFS. Therefore, the results suggest mathematical features of cancer cell groups could indeed have important and independent impact on the clinical outcomes of BC.

Of particular interest is the result of multivariate analysis, which showed that TNs integrated parameter 1 had higher predictive effectiveness on 5-DFS than N stage and hormone receptor status. The practical importance of this finding is that in the case of early BC where lymph nodes involvement has not occurred, there could be other alternative parameters obtained from the local tumor itself to help predict the aggressive behaviors of BC, and such parameters could be as useful as the lymph nodes information.

Another interesting finding from multivariate analysis is that the predictive effectiveness of integrated parameter 1 is lower than the currently adopted WHO histological grading system, which is based on cancer cell groups information-tubule formation, individual cancer cell proliferation information-mitotic activity, and cancer cell nucleus information-nuclear pleomorphism [10]. Therefore, new information on TNs could not substitute for the WHO grading system, but rather it could be supplementary to the system.

The limitations of this study should be admitted, as in the following 3 aspects. Firstly, we extracted prognostic factors only from morphologic characteristic of TNs, but did not explore the role of mitotic activity, individual cancer cell morphology, individual unclear pleomorphism, and tumor microenvironment, which are all related to prognosis [33]–[35]. Secondly, we evaluated the relationship between MPs and prognosis of patients by DFS rather than overall survival. Lastly, although the construction of TMAs was strictly according to a criterion that only tumor tissues at the cancer invasion front were selected, not every core of TMAs could completely represent the whole tumor. Therefore, the results from this study should be interpreted with caution and future validation work with larger sample size and longer clinical follow-up is warranted.

Nevertheless, this study demonstrated that mathematic features of TNs morphology could help predict the biological behaviors of breast IDC, and the predictive importance of TNs integrated parameter 1 could be no less than N stage, but no more than histological grading. Therefore, for future work to develop a comprehensive model to predict IDC invasion and metastasis based on information extracted from local tumor itself, the integrated parameter 1 combining TNs number, circularity and total perimeter could be considered as useful candidates to be incorporated into this strategy.
